# Effect of PVP Concentration on the Crystalline Structure and Morphology of Hydroxyapatite via Microwave-Assisted Hydrothermal Synthesis

**DOI:** 10.3390/ma19020223

**Published:** 2026-01-06

**Authors:** Lesly S. Villaseñor-Cerón, Demetrio Mendoza-Anaya, Andres Galdámez-Martínez, Claudia E. Gutiérrez-Wing, Omar A. Domínguez-Ramírez, Josué E. Muñoz-Pérez, Ventura Rodríguez-Lugo

**Affiliations:** 1 Área Académica de Ciencias de la Tierra y Materiales, Universidad Autónoma del Estado de Hidalgo, Mineral de la Reforma 42184, Hidalgo, Mexico; lesly_villasenor@uaeh.edu.mx (L.S.V.-C.); josue_munoz@uaeh.edu.mx (J.E.M.-P.); 2 Instituto Nacional de Investigaciones Nucleares, Ocoyoacac 52750, Estado de México, Mexico; demetrio.mendoza@inin.gob.mx (D.M.-A.); claudia.gutierrez@inin.gob.mx (C.E.G.-W.); 3 Nano and Molecular Systems Research Unit, University of Oulu, 90014 Oulu, Finland; andres.galdamezmartinez@oulu.fi; 4 Área Académica de Computación y Electrónica, Universidad Autónoma del Estado de Hidalgo, Carr. Pachuca-Tulancingo Km. 4.5, Mineral de la Reforma 42184, Hidalgo, Mexico; omar@uaeh.edu.mx

**Keywords:** microwave, hydroxyapatite, polyvinylpyrrolidone, surfactant, biomaterial

## Abstract

**Highlights:**

**What are the main findings?**

- Rietveld refinement analysis confirms the formation of the crystalline Hexagonal Phase of the monoclinic structure.
- The use of PVP influences the formation of the crystalline phases of Hap.
- The addition of PVP at low concentrations (0.1 and 0.2%wt) induces dimensional and morphological instability of Hap. At a PVP concentration of 0.3%wt, dimensional and morphological stability is restored, with nanorods being the predominant morphology.

**What are the implications of the main findings?**

- The results indicate that PVP acts as an effective surfactant for achieving dimensional and morphological control of hydroxyapatite.
- The formation of well-defined nanorod morphologies at 0.3%wt PVP establishes a valuable reference for future studies on the controlled synthesis of Hap nanostructures.
- The proposed model provides insight into the interactions between PVP and Hap, contributing to the advancement of research on the dimensional and morphological control of nanomaterials. This understanding supports the design of materials with optimized properties for targeted applications, including biomaterials.

**Abstract:**

In this study, hydroxyapatite was synthesized using a microwave-assisted hydrothermal method. Calcium nitrate tetrahydrate (Ca(NO_3_)_2_·4H_2_O) and ammonium phosphate ((NH_4_)_2_HPO_4_) served as precursors in a pH 10 ammonium hydroxide (NH_4_OH) solution. Polyvinylpyrrolidone (PVP) was employed as a surfactant at varying concentrations of 0 (M0), 0.1% (M1), 0.2% (M2), and 0.3%wt (M3) to control particle size and morphology. The synthesized samples were characterized using Transmission Electron Microscopy (TEM), Scanning Electron Microscopy (SEM), X-ray Diffraction (XRD) and Fourier Transform Infrared Spectroscopy (FTIR). The addition of PVP during synthesis resulted in Ca/P ratios ranging from 0.93 to 1.37, and promoted predominantly rod-like morphologies. Samples M1 and M3 exhibited average diameters of 11.23–104.24 nm and lengths of 47.21–222.32 nm. XRD analysis confirmed the presence of both hexagonal and monoclinic phases, with crystallite sizes varying from 18.66 to 22.49 nm. FTIR spectra of sample M1 revealed an elongation at 3432 cm^−1^ corresponding to OH^−^ groups, indicative of water absorption within the material structure. Vibrational bands at 2950–2300, 1090, and 975 cm^−1^, attributed to C–H bonds in PVP were also identified. These findings highlight the influence of PVP concentration on the structural and morphological properties of hydroxyapatite, providing insights into its potential applications in various fields.

## 1. Introduction

Hydroxyapatite (Hap) is a bioceramic material with characteristics and properties very similar to those of the main components of bone. Stoichiometrically, it presents a Ca/P ratio of 1.67, which confers chemical stability and excellent biocompatibility [1], osteointegration [2], bioactivity [3], osteoconduction [4], thermoluminescence [5], among other properties.

Likewise, depending on the Ca/P ratio, when it is lower or higher than 1.67, solubility increases or decreases accordingly, promoting gradual degradation in biological environments, which allows for the production of bioceramics with high or low solubility [6,7]. Bioabsorbable bioceramics are those that have a Ca/P ratio between 1.0 and 1.67, which favors gradual degradation in biological environments, contributing to bone tissue regeneration as they are absorbed. These characteristics make them potentially useful for the development of scaffolds in tissue engineering, as they provide temporary support while natural tissue regeneration occurs. On the other hand, bioceramics with a Ca/P ratio < 1.0 exhibit high solubility and low chemical stability, making them useful for the development of biomaterials. The ideal Ca/P ratio is 1.67–1.70, at which an increase in osteogenic processes is observed. In contrast, bioceramics with a Ca/P ratio > 1.70 exhibit minimal bioabsorption in biological environments, making them suitable for applications that require long-term durability, such as implants, bone grafts, and coatings [8,9,10,11,12,13].

Hap can be synthesized using various methods, including dry, wet, and high-temperature processes. Among the wet-chemical techniques, the microwave-assisted hydrothermal method involves a limited number of steps, generating fewer by-products. It is a fast, uniform, and effective method that allows the reaction kinetics to be increased by one or two orders of magnitude, producing structures with controlled size distribution. However, precise control of morphology is not always achieved [14,15,16,17,18]. To address this, numerous studies have explored the use of surface-active agents to regulate the size and shape of the synthesized materials [15], such as anionic, cationic, amphoteric, non-ionic and polymeric surfactants [19]. One such polymeric surfactant is polyvinylpyrrolidone (PVP), a synthetic polymer known for its excellent biocompatibility and low toxicity. Its structural similarity to proteins facilitates its application in biomedical fields, especially in dental implants and bone grafts [20]. PVP’s unique chemical structure includes highly polar amide groups, imparting hydrophilic and polar attraction properties, as well as non-polar methylene groups that confer hydrophobic characteristics. It also contains two electron-donation centers (nitrogen and oxygen in the polar groups), with the oxygen in the C=O group being the most favorable interaction site due to steric constraints on the nitrogen atom [21,22]. Recent research highlights PVP’s role in Hap synthesis, enabling the production of nanostructures with controlled morphologies. For example, Govindan Suresh Kumar et al. reported the microwave-assisted synthesis of Hap nanofibers at 200 °C for 20 min using Ca(NO_3_)_2_·4H_2_O and K_2_HPO_4_ as precursors, adjusted to pH 10. They achieved a hexagonal crystalline phase with nanofiber diameters of 10 nm and a Ca/P ratio between 1.73 and 1.78 [23]. Similarly, C. F. Qiu et al. synthesized spherical Hap structures (30–50 nm) using a biomimetic method and varying PVP concentrations (0–5%) in a solution at pH 10.5 [24]. Additionally, Yanjie Zhang et al. employed a biomimetic process at 60 °C for 2–5 days, using Ca(NO_3_)_2_·4H_2_O and H_3_PO_4_ as precursors (pH 3), with PVP to control Hap nucleation and crystal growth. They obtained rod-like structures with a hexagonal crystalline phase and preferential growth along the <002> direction [25]. Youssef Guesmi et al. synthesized a Hap/PVP composite using CaCl_2_ and Na_2_HPO_4_ at ambient temperature for 72 h at pH 10 via a wet chemical process. Their results showed a reduction in crystal growth along the (002) plane and lower crystallinity, attributed to the interaction between the OH^−^ groups of Hap and the C=O bond of PVP. This composite demonstrated potential for bone graft applications [20]. Lastly, Xingwei Du et al. synthesized Hap nanorods (20–25 nm) via a hydrothermal method at 180 °C for 24 h, reporting a hexagonal phase and a Ca/P ratio of 1.5, with PVP promoting crystalline growth along the c-axis [26]. As noted in these reports, PVP can be used to regulate the morphology of synthetic Hap. However, further experimental studies on synthesis conditions and PVP concentration are necessary to achieve greater control over the final characteristics of Hap.

Therefore, this work aims to investigate the influence of PVP concentration on the crystalline structure and morphology of Hap synthesized via a microwave-assisted hydrothermal method, contributing to the dimensional and morphological control of advanced materials with great potential for biomaterials development. Although the biological properties of the material were not evaluated in this work, the literature indicates that the incorporation of PVP does not compromise the biological properties of Hap, maintaining its biocompatibility and cell viability, thereby supporting its potential application in biomaterials development [27,28,29,30,31].

## 2. Materials and Methods

### 2.1. Materials

For the synthesis, calcium nitrate tetrahydrate, CAS: No. 13477-34-4, (Ca(NO_3_)_2_·4H_2_O) and ammonium dihydrogen phosphate, CAS; No. 287488-11-3 [(NH_4_)_2_HPO_4_] from Meyer (Vallejo, CA, USA) were used as precursors. The pH was adjusted with ammonium hydroxide, CAS: No. 1336-21-6 (NH_4_OH) 28.0–30.0% (Ammonia) from Meyer. Polyvinylpyrrolidone (PVP), CAS: No. 9003-39-8 [(C_6_H_9_NO)_n_] with an average molecular weight of 10,000 g/mol was purchased from Sigma-Aldrich (Toluca, Mexico).

### 2.2. Hydroxyapatite-Polyvinylpyrrolidone Synthesis

The Hap-PVP samples were synthesized by dissolving Ca(NO_3_)_2_·4H_2_O and (NH_4_)_2_HPO_4_ in 40 mL of distilled water. Subsequently, the PVP surfactant was dissolved in 10 mL of distilled water and dispersed using ultrasound at a power of 5W for 4 min. The precursor solution of (NH_4_)_2_HPO_4_ was then added to the Ca(NO_3_)_2_·4H_2_O precursor, followed by the dropwise addition of the PVP surfactant at varying concentrations (0%wt [M0], 0.1%wt [M1], 0.2%wt [M2] and 0.3%wt [M3]). Next, NH_4_OH was dropwise added to the solution to adjust the pH to 10. The resulting Hap samples were then transferred to a Teflon reactor with a maximum capacity of 100 mL, filling one-quarter of the reactor, and subjected to microwave-assisted synthesis under programmed conditions: 800 W of power for 10 min to reach a temperature of 200 °C, followed by maintaining the reaction at that temperature for 30 min. Upon completion, the sediment was washed three times with distilled water, dried at 100 °C for 24 h, and ground into a fine powder. Finally, the samples were calcined at 500 °C for 3 h.

### 2.3. Characterization

To determine the morphology and particle size of the Hap samples, a Jeol JSM-5900LV Scanning Electron Microscope (SEM) (Tokyo, Japan) equipped with an X-ray energy dispersive spectrometer (EDS) was used. Before EDS analysis, a small amount of sample was released on the aluminum sample holder. Elemental chemical results were obtained from three different zones of each sample. SEM equipment was operated in high vacuum mode, at 20 KV. X-ray diffraction (XRD) analysis was performed in a Diffractometer D8 Discover Bruker (Radiation source CuKα = 1.5406 Å) operating at 40 kV and 40 mA (Billerica, MA, USA). Diffraction patterns were collected from 10.0° to 70.0° in 2θ scale, with a step size of 0.03°. Using the Match! 3 and Fullprof programs, a Rietveld refinement analysis was carried out. FTIR analysis was made in a Brand: Perkin Elmer FTIR System Spectrum Gx (Waltham, MA, USA). The average particle size was calculated using ImageJ 1.50i software. A detailed morphological analysis was performed in a Jeol JEM 2010HT transmission electron microscope (TEM) (Tokyo, Japan). Samples were prepared by dispersing the samples in ethanol and depositing a drop on a carbon-coated copper grid.

## 3. Results and Discussion

### 3.1. Scanning Electron Microscopy

**Figure 1:**
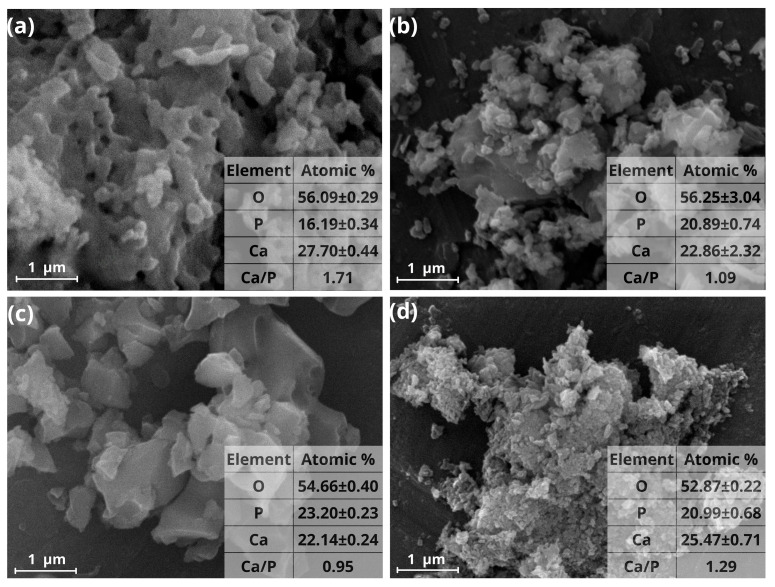
SEM micrographs of Hap samples with different PVP concentrations: (**a**) M0, (**b**) M1, (**c**) M2 and (**d**) M3.

Figure 1a corresponds to the SEM micrograph of M0 sample in which agglomerates consisting of bar-shaped structures with particle sizes of 32 nm width and 81 nm in length can be visualized. Figure 1b,c correspond to a SEM micrograph of M1 and M2 samples where agglomerates of particles from fractions to units of micrometers are observed; some of these particles showed flat faces while other present spherical morphology. In the case of M3 sample (Figure 1d) agglomerates with sizes ranging from 200 to 400 nm and granular appearance were observed.

As the concentration of PVP increases, there is a change in the morphology of the material that goes from the formation of bars in M0 sample, agglomerates of flat and spherical particles in M1 and M2 samples and agglomerates with granular appearance in M3 sample.

Similarly, in the bottom right corner of each micrograph shown in Figure 1, the Energy Dispersive Spectroscopy (EDS) analysis is presented and was used to identify the Ca/P ratio for each sample. For pure Hap (sample M0), a Ca/P ratio of 1.71 was obtained, classifying it as an insoluble bioceramic, while samples M1 and M3 present Ca/P ratios of 1.09 and 1.29, respectively, which are associated with bioabsorbable bioceramics. Sample M2 also falls into this category, presenting a Ca/P ratio of 0.95, close to 1. Based on these results, the synthesized samples are suitable for the development of biomaterials, where the specific application depends on the type of bioceramic used, including bone regeneration, implants, bone fillers, and scaffolds for tissue engineering, among others.

### 3.2. X-Ray Diffraction

**Figure 2:**
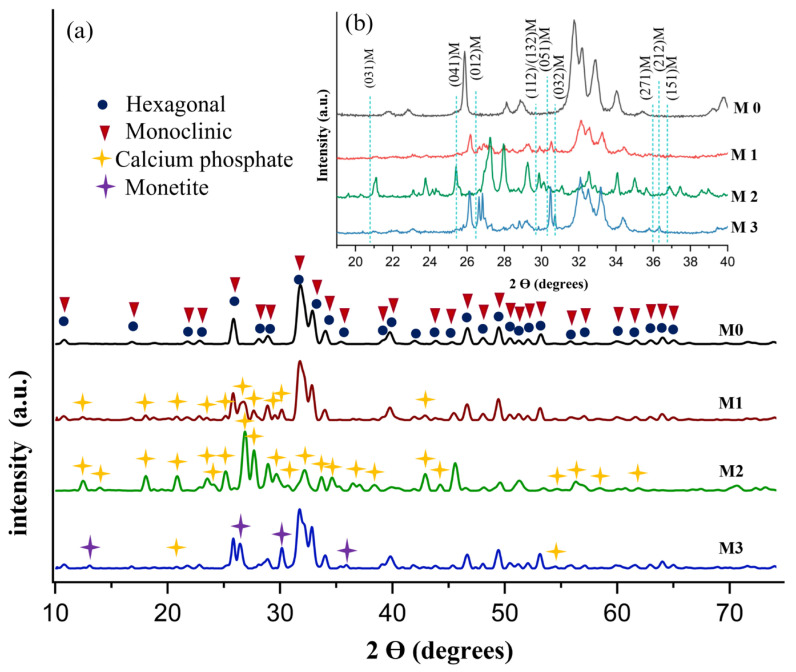
X-ray diffractogram (**a**) Hap samples with different PVP concentrations and (**b**) enlargement of the diffractogram in the range of 20.0° to 40.0° in 2θ.

X-ray diffractogram of pure Hap and Hap-PVP samples is shown in Figure 2. Peaks at 10.8, 16.8, 21.8, 22.9, 25.8, 28.1, 28.9, 31.7, 32.9, 34.0, 35.4, 39.2, 39.8, 42.0, 43.8°, 45.3°, 46.7°, 48.1°, 49.4°, 50.4°, 51.2°, 52.1° and 53.1° in 2-theta (degree) are appreciated, which correspond to the main planes (100), (101), (200), (111), (002), (102), (210), (211), (300), (202), (301), (212), (310), (311), (113), (203), (222), (312), (213), (321), (410), (402) and (004) of hexagonal hydroxyapatite with index card 09-0432, according to ICDD (International Centre for Diffraction Data). Low-intensity diffraction peaks at 55.8°, 57.1°, 60.4°, 61.6°, 63.0°, 64.0°, 65.0° and 66.3° at 2θ, correspond to the planes (322), (313), (420), (214), (502), (323), (511) and (413), respectively, which were also associated with the hexagonal crystalline phase of the Hap. In addition, it was possible to observe low intensity diffraction peaks at 55.9°, 57.1°, 60.3°, 61.5°, 62.9°, 64.1°, 64.9° and 66.3° in 2-theta (degree) which were associated with the planes (034), (−214), (−3 12 1), (281), (501), (−2 10 1), (521) and (−4 12 2), respectively, corresponding to the monoclinic Hap according to index card 76-0694. Inset in Figure 2 shows an enlargement of the diffractogram in the range from 20.0° to 40.0° in 2θ to allow identify the prohibited reflections for the hexagonal phase in 20.7°, 25.3°, 26.4°, 29.7°, 30.2°, 30.7°, 35.9°, 36.2° and 36.7° in 2θ corresponding also to the monoclinic crystalline planes (031), (041), (012), (112)/(132), (051), (032), (271), (212) and (151) of Hap, respectively, as reported by J. Reyes-Gasga et al. [32]. This result is noteworthy because, in the consulted literature where PVP was used as surfactant, the monoclinic phase was considered absent, despite its greater stability than the hexagonal phase. Therefore, the analysis confirms the coexistence of hexagonal and monoclinic crystalline phases in the synthesized samples and the formation of planes (041), (032) and (212) associated with the increasing concentration of PVP.

Likewise, the formation of secondary phases such as Calcium Phosphate (PDF 09-0345), present at 12.43°, 13.90°, 18.44°, 20.76°, 23.56°, 24.06°, 25.24°, 26.91°, 27.70°, 29.67°, 30.74°, 32.33°, 33.58°, 34.68°, 36.47°, 38.34°, 42.87°, 44.35°, 54.56°, 56.40°, 58.43°, 61.87° and Monetite (PDF 09-0080) in 13.02°, 26.41°, 30.07° and 35.88 was detected. Based on the literature, these secondary phases do not affect the biomaterial properties of Hap, because calcium phosphate and monetite are also used within the medical field [33,34]. Specifically, the cytotoxicity tests in monetite demonstrate its non-toxicity and it has been suggested as a bone graft material for hard tissue repair and regeneration.

Using the Debye–Scherrer equation and the diffractograms shown in Figure 2, the average crystallite size was calculated from the main planes (002), (211), and (300), considering K = 0.94. Table 1 presents the crystallite size and the Rietveld refinement analysis corresponding to the percentage of hexagonal and monoclinic crystalline phases of each sample, based on the indexing cards 96-901-4314 and 96-202-0365 for the hexagonal and monoclinic phases, respectively. It can be observed that the crystallite size changes depending on the PVP concentration. Likewise, the Rietveld refinement confirms the presence of both crystalline phases. Pure Hap exhibits the smallest crystallite size, whereas sample M3 shows the largest crystallite size.

### 3.3. Fourier Transform Infrared Spectroscopy

**Figure 3:**
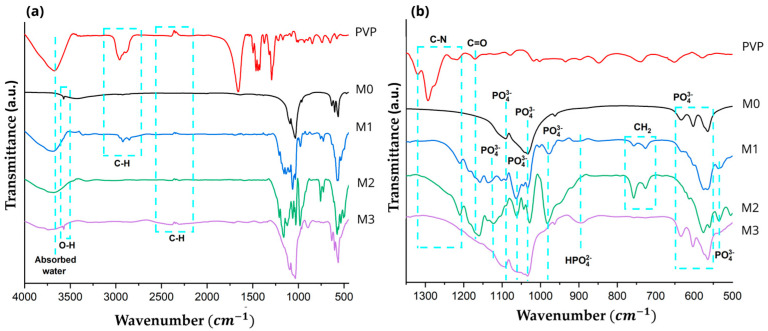
IR spectrum of (**a**) PVP and Hap samples with different PVP concentrations: M0, M1, M2 and M3, (**b**) Enlargement of the spectrum in the range of 500 cm^−1^ to 1300 cm^−1^ in wavenumber.

FTIR analysis was conducted to examine the chemical structure of Hap and Hap-PVP, with the spectra presented in Figure 3. In Figure 3a, the M1 sample exhibited an elongated OH^-^ band at 3432cm^−1^, indicating water absorption within the material structure, while bands at 1638 and 1317 cm^−1^ are associated with the stretching vibrations of absorbed water in hydroxyapatite [20,35]. For M1, M2 and M3 samples, absorption bands between 2950–2300 and 975 cm^−1^ are observed, corresponding to the vibrational modes of asymmetric stretching of the C-H bond [20,35,36]. That is, although a calcination process was carried out, remnants of the surfactant are still present. Likewise, Figure 3b presents an enlarged view of the wavenumber range from 500 to 1300 cm^−1^, where the characteristic absorption bands of Hap were identified at 1210, 983, 962, 635, and 493 cm^−1^, corresponding to the vibrational stretching modes of PO_4_^3−^ groups [25,26,37]. Additional bands at 1030–1020 cm^−1^ and 602, 563 and 528 cm^−1^ were assigned to the vibrational modes of asymmetric stretching and bending of PO_4_^−3^ ions, respectively [25]. Likewise, the band observed at 895 cm^−1^ is attributed to the HPO_4_^2−^ group, corresponding to the bending vibrational mode of the P–O bond. Similarly, the bands observed at 755 and 727 cm^−1^ are associated with C–H bonds. Bands in the 800–700 cm^−1^ region were attributed to the bending vibrational mode of PO_4_^3−^, characteristic of Hap [38,39].

### 3.4. Transmission Electron Microscopy

**Figure 4:**
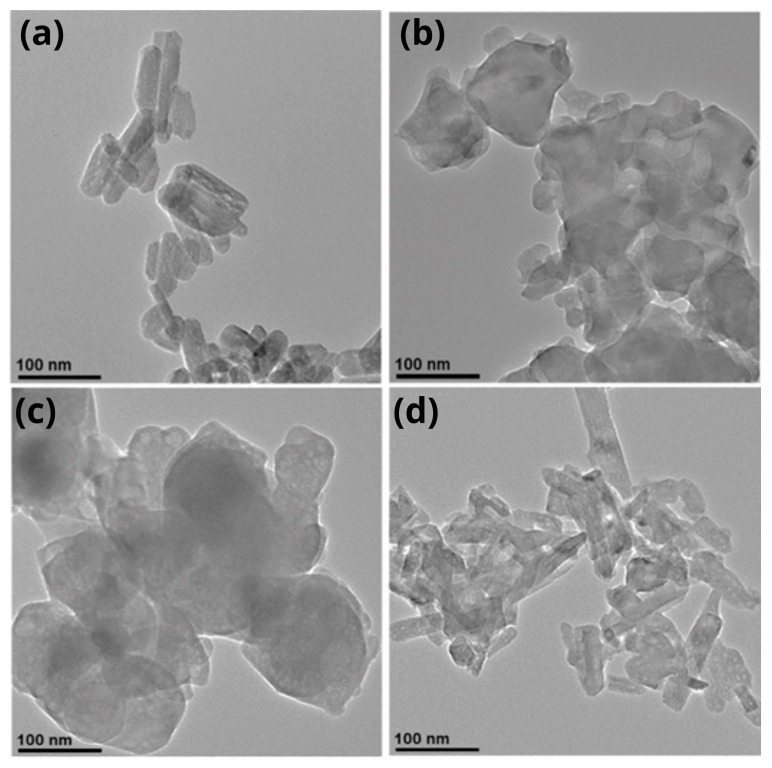
TEM micrographs of Hap samples with different PVP concentrations: (**a**) M0, (**b**) M1, (**c**) M2 and (**d**) M3.

A detailed analysis of the morphology and particle size of pure Hap and Hap-PVP samples was performed by Transmission Electron Microscopy and is presented in Figure 4. The TEM micrograph of M0 is shown in Figure 4a, in which two distributions of nanobars can be distinguished: one with an average width of 23 nm and length of 46 nm, and a second with an average width of 32 nm and length of 82 nm. In Figure 4b (M1), anhedral particles with a lamellar appearance are observed, with a size distribution ranging from approximately 23 nm to 130 nm. Similar structures are seen in the M2 sample (Figure 4c), although the particle size is larger, reaching up to 215 nm. Finally, in the micrograph of the M3 sample (Figure 4d), the two characteristic Hap morphologies—nanobars and nanosheets—are observed, with sizes of 21 nm width and 45 nm length for nanobars, and 32 nm width and 117 nm length for nanosheets. It should be noted that nanosheet structures are less abundant.

**Figure 5:**
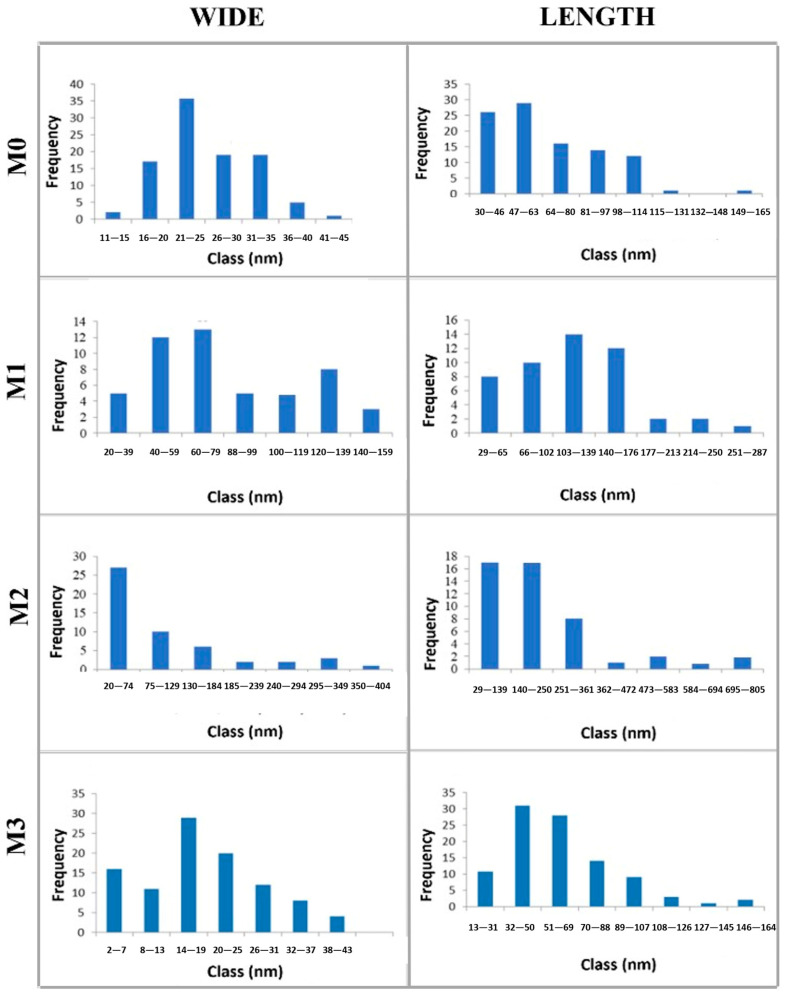
Size distribution of Hap samples with different PVP concentrations: M0, M1, M2 and M3.

On the other hand, using TEM micrographs, a statistical analysis of the size of 100 particles from each sample was carried out and is presented in the histograms in Figure 5. Table 2 shows the arithmetic mean and standard deviation for each sample. According to these data, the Hap sample synthesized with the highest PVP content exhibits the lowest average width and length.

As well as in the SEM results, when the concentration of PVP increases, there is a change in the morphology of the Hap that goes from the formation of bars in pure Hap (M0) sample, anhedral particles with lamellar appearance in M1 and M2 samples and mainly nanobars in M3 sample.

Table 3 presents relevant information, showing the use of different synthesis routes, precursors, and experimental conditions. In all cases, the synthesis of Hap with various morphologies and a wide range of particle sizes is reported. In particular, the microwave-assisted hydrothermal method has demonstrated the production of crystalline Hap with nanostructured features in short synthesis intervals, establishing the influence of temperature and reaction time on the formation of agglomerates into well-defined nanorod structures within a temperature range of 60 to 180 °C and reaction times of 10 to 50 min. This is consistent with the results of the present study, where it is observed that the type of microwave heating employed promotes the formation of nanostructured Hap. On the other hand, despite the scarcity of specific articles on the use of PVP in the synthesis of Hap by the microwave-assisted hydrothermal method, it can be indicated that surface-active agents like PVP promote dimensional and morphological control of Hap, yielding well-defined structures with a hexagonal crystalline structure. This contrasts with our results, in which we report the presence of hexagonal and monoclinic Hap with nanobar or anhedral morphologies depending on the PVP concentration, using 200 °C for 30 min. Additionally, we report the presence of by-products such as monetite, which has also been suggested as a biomaterial.

**Figure 6:**
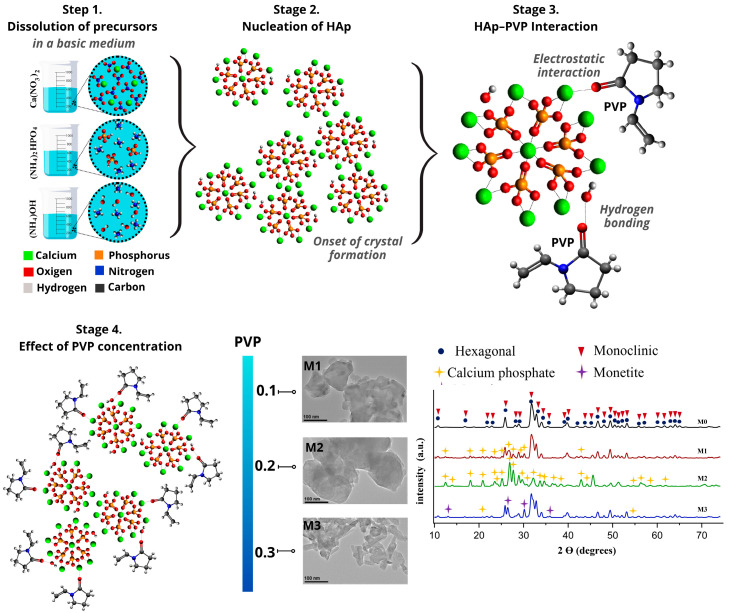
Morphological transformation of Hap at concentrations of 0 (M0), 0.1 (M1), 0.2 (M2) and 0.3 (M3) % by weight of PVP.

Figure 6 shows the stages of Hap formation. In stage 1, the dissolution of the precursors is represented, leading to the formation of reactive calcium and phosphate species in a basic medium. The high pH favors the formation of deprotonated phosphate species, facilitating Hap formation. In stage 2, the nucleation and crystal growth of Hap are described. When the system reaches sufficient energy, the nucleation process begins, during which the reactive species from the precursors, as well as OH^−^ groups from the medium, are incorporated into the HAp crystal lattice. In stage 3, the interaction between Hap and PVP as a surfactant is illustrated. PVP consists of a hydrophilic head corresponding to the pyrrole group, which carries a partial negative charge, and a hydrophobic tail corresponding to the alkyl chain. It is classified as a non-ionic surfactant because the hydrophilic pyrrole group contains functional groups such as C=O that carry a partial negative charge. This allows interaction with Ca^2+^ ions through electrostatic attractions, as well as hydrogen bonding with the OH^−^ groups of Hap. In stage 4, it is observed that when nucleation and crystal growth of Hap occur in the presence of PVP, the growth of the crystallites can be limited. At low PVP concentrations (M1 and M2), disordered growth is observed, which can be attributed to heterogeneous interactions between PVP and the crystalline formation of Hap, whereas at higher PVP concentrations (M3), a more homogeneous coating occurs during crystal growth, allowing uniform control of HAp size and shape [20,24,25,44].

The incorporation of PVP in the synthesis of Hap at concentrations of 0.1% and 0.2% by weight (M1 and M2) causes destabilization due to the interaction of the surfactant with Hap, leading to the formation of anhedral structures attributed to secondary phases such as calcium phosphate. During microwave-assisted hydrothermal synthesis, temperature gradients are generated, promoting uneven heating. Additionally, the basic medium contributes to the formation of Ca(OH)_2_, which, when reacting with phosphate species, acts as a transient phase for Hap formation. Incorporating PVP at low concentrations allows interactions to occur during the nucleation and crystal growth stage through chemical interactions and steric effects, creating a barrier on the Hap crystal surface that prevents calcium and phosphate species from continuing to bond for material growth, contributing to more transient-phase species (calcium phosphates) remaining unreacted. Subsequently, during the calcination process, thermal dehydration occurs, promoting the formation of monetite from poorly crystallized calcium phosphates [45,46]. This observation is consistent with the results obtained from FTIR and XRD, where a greater formation of bands and crystalline planes corresponding to the PO4^3−^ group and secondary phases, respectively, was detected. At a PVP concentration of 0.3wt% (M3), it is observed that the Hap morphology begins to stabilize, exhibiting a predominant bar-like morphology similar to that of sample M0, demonstrating that the incorporation of PVP affects the morphology and size of the material.

## 4. Conclusions

In the present research, the conditions and reaction parameters suitable for the synthesis of Hap using the microwave-assisted hydrothermal method were established. Experimental results showed an appreciable influence of PVP concentration on the morphological and crystalline characteristics of the synthesized hydroxyapatite. It was mainly observed that nanobars grew with widths ranging from 11.23 to 104.24 nm and lengths from 64.82 to 222.32 nm. Likewise, the formation of both hexagonal and monoclinic crystalline phases was identified, a particularly significant result, as the consulted literature typically reports only the presence of the hexagonal phase. Based on the present research, nanobars synthesized at a PVP concentration of 0.3wt% exhibited the best characteristics in terms of particle shape and size control, with average sizes of 11.23 nm in width and 47.21 nm in length.

Furthermore, the presence of monetite in the synthesized Hap samples opens new avenues, given its application as a bone graft material. Therefore, based on these results, this work presents PVP as a promising morphological and structural regulator in the synthesis of hydroxyapatite nanomaterials, thereby broadening its biomedical applications.

## Figures and Tables

**Table 1:** Crystallite size and rietveld refinement of Hap samples with different PVP concentrations: M0, M1, M2 and M3.

Samples	Crystallite Size (nm)			Rietveld Refinement	
	Hap	Calcium Phosphate	Monetite	Hexagonal (%)	Monoclinic (%)
M0	18.22	-------	-------	15.50	84.60
M1	21.08	21.20	-------	88.90	11.10
M2	18.66	21.00	-------	74.10	25.90
M3	22.49	-------	28.87	88.80	11.20

**Table 2:** Arithmetic means and standard deviation of width and length for Hap samples with different PVP concentrations: M0, M1, M2 and M3.

Sample	Average Value	
	Width (nm)	Length (nm)
M0	24.94 ± 6.31	64.82 ± 24.89
M1	80.78 ± 36.36	119.36 ± 52.31
M2	104.24 ± 89.73	222.32 ± 180.90
M3	11.23 ± 9.50	47.21 ± 28.30

**Table 3:** Summary of the synthesis of Hap in the presence of PVP.

Synthesis Method	Conditions	Precursors	Surfactant Concentration PVP	Crystalline Phase	Morphology and Size	Ref.
Biomimetic Method	2–7 days to 60 °C, pH 3 (HNO_3_)	Ca(NO_3_)_2_·4H_2_O H_3_PO_4_	0.011 and 0.14 mmol	Hexagonal	Nanobars Diameter: 10–20 nm Longitude: 250–300 nm	[25]
Biomimetic method	pH 10.5	Ca(NO_3_)_2_·4H_2_O (NH_4_)_3_PO_4_·3H_2_O	0–5%	Hexagonal	Spheres 30–50 nm	[24]
Wet chemistry	room temperature for 72 h to a pH 10	CaCl_2_ Na_2_HPO_4_	3.33 g	Hexagonal	Regular morphologies 59–280 nm	[20]
Hydrothermal method	180 °C 24 h	Na_2_HPO_4_·12H_2_O Ca(NO_3_)_2_·4H_2_O	2.5, 9 y 10^−4^ mol/L	Hexagonal	Nanobars Diameter: 20 a 25 nm	[26]
Microwave-assisted hydrothermal	100–140 °C 30 min	Ca(NO_3_)_2_·4H_2_O H_3_PO_4_	---	Hexagonal	Agglomerated and nanorods	[40]
Microwave-assisted hydrothermal	60–180 °C 50 min	CaCl_2_·2H_2_O Na_2_HPO_4_·12H_2_O	---	---	Diameter: 19–30 nm Longitude: 50–700 nm	[41]
Microwave-assisted hydrothermal	80 °C 30 min	Ca(NO_3_)_2_·4H_2_O (NH_4_)_3_PO_4_·3H_2_O	---	Hexagonal	Nanobars Diameter: 18 a 80 nm	[42]
Microwave-assisted hydrothermal	80 °C 10 min	Ca(OH)_2_ H_3_PO_4_	---	Hexagonal	Agglomerated	[43]
Microwave-assisted hydrothermal	200 °C 20 min pH 10	Ca(NO_3_)_2_·4H_2_O K_2_HPO_4_	1 g	Hexagonal	Nanofibers 10 nm (Diameter)	[23]

## Data Availability

The original contributions presented in this study are included in the article. Further inquiries can be directed to the corresponding author.

## Abbreviations

The following abbreviations are used in this manuscript:

Hap	Hydroxyapatite
PVP	Polyvinylpyrrolidone
TEM	Transmission Electron Microscopy
SEM	Scanning Electron Microscopy
XRD	X-ray Diffraction
FTIR	Fourier Transform Infrared Spectroscopy
nm	Nanometer
